# Passive immunotherapy for adults hospitalized with COVID-19: An individual participant data meta-analysis of six randomized controlled trials

**DOI:** 10.1371/journal.pmed.1004616

**Published:** 2025-07-07

**Authors:** Kirk U. Knowlton, Lianne K. Siegel, Christina E. Barkauskas, Sanjay Bhagani, Nila J. Dharan, Edward M. Gardner, Robert L. Gottlieb, Marie Helleberg, Helene C. Highbarger, Thomas L. Holland, Christian Kjer Heerfordt, Susana Lazarte, Lindsey M. Leither, Joseph Lutaakome, Magdalena Ardelt, Eleftherios Mylonakis, Sean W. X. Ong, Jeffrey Scott Overcash, Hassan Taha, Phyllis C. Tien, Barbara W. Trautner, David Vallee, Amy C. Weintrob, Giota Touloumi, Abdel G. Babiker, for the STRIVE ACTIV-3/TICO Study Group

**Affiliations:** 1 Intermountain Heart Institute, Intermountain Medical Center, Salt Lake City, Utah, United States of America; 2 Division of Biostatistics and Health Data Science, School of Public Health, University of Minnesota, Minneapolis, Minnesota, United States of America; 3 Department of Medicine, Division of Pulmonary and Critical Medicine, Duke Medicine, Durham, North Carolina, United States of America; 4 Royal Free Hospital and University College, London, United Kingdom; 5 Kirby Institute, University of New South Wales Sydney, Sydney, New South Wales, Australia; 6 Public Health Institute at Denver Health, Denver Hospital Authority, Denver, Colorado, United States of America; 7 Baylor University Medical Center, Baylor Scott & White The Heart Hospital, Dallas, Texas, United States of America; 8 Department of Infectious Diseases, Rigshospitalet, Copenhagen University Hospital, Copenhagen, Denmark; 9 Virus Isolation and Serology Laboratory, Frederick National Laboratory, Applied and Developmental Directorate, Frederick, Maryland, United States of America; 10 Department of Medicine, Division of Infectious Diseases, Duke University, Duke Clinical Research Institute, Durham, North Carolina, United States of America; 11 Department of Respiratory Medicine and Infectious Diseases, Copenhagen University Hospital - Bispebjerg and Frederiksberg, Copenhagen, Denmark; 12 Division of Infectious Diseases and Geographic Medicine, Department of Internal Medicine, University of Texas Southwestern Medical Center, Dallas, Texas, United States of America; 13 Intermountain Medical Center, Salt Lake City, Utah, United States of America; 14 Medical Research Council/Uganda Virus Research Institute and London School of Hygiene and Tropical Medicine Uganda Research Unit, Entebbe, Uganda; 15 Ralph H Johnson VA Medical Center, Charleston, South Carolina, United States of America; 16 Department of Medicine, Houston Methodist Hospital & Houston Methodist Academic Institute, Houston, Texas, United States of America; 17 University of Toronto, Toronto, Canada; 18 National Centre for Infectious Diseases, Singapore, Singapore; 19 Velocity Clinical Research, San Diego, California, United States of America; 20 Stormont Vail Health, Vail, Colorado, United States of America; 21 University of San Francisco, San Francisco, California, United States of America; 22 Department of Medicine, Baylor College of Medicine, Houston, Texas, United States of America; 23 Frederick National Laboratory for Cancer Research/Leidos Biomedical Research, Frederick, Maryland, United States of America; 24 Washington D.C. Veterans Affairs Medical Center and George Washington University, Washington, DC, United States of America; 25 Department of Hygiene, Epidemiology, & Medical Statistics, Medical School, National & Kapodistrian University of Athens, Athens, Greece; 26 University College London, London, United Kingdom; Erasme University Hospital, BELGIUM

## Abstract

**Background:**

Anti-SARS-CoV-2 monoclonal antibodies (mAb) reduce the risk of hospitalization in outpatients with mild-to-moderate COVID-19. However, the efficacy of treatment with mAbs and other passive immunotherapies in patients hospitalized with severe COVID-19 is not clear. The objective of this study was to assess the clinical effect of passive immunotherapy and its heterogeneity according to baseline endogenous neutralizing antibody status and SARS-CoV-2 antigen level, in adults hospitalized with SARS-CoV-2 infection and severe COVID-19.

**Methods and findings:**

We carried out a two-stage individual participant data meta-analysis of six double-blind, randomized, placebo-controlled trials conducted under the Therapeutics for Inpatients with COVID-19 (TICO) and the similarly designed Inpatient Treatment with Anti-Coronavirus Immunoglobulin (ITAC) master protocols. Within each trial, three major outcomes (sustained recovery, mortality, and a composite safety outcome) were compared between treatment and placebo using Fine-Gray and Cox proportional hazards models. Trial-specific treatment differences for each of the three outcomes were pooled using a common effect meta-analysis. A total of 3,079 patients hospitalized for COVID-19 were enrolled in the six trials. Only 18% had received at least one dose of an anti-SARS-CoV-2 vaccine. Overall, the median plasma SARS-CoV-2 antigen level was 1,421 (IQR: 231−4,568) pg/mL, and 51% of patients were endogenous neutralizing antibody positive at study entry. The overall summary estimate of sustained recovery rate ratio (RRR) of the treatment versus placebo group was 1.06 (95% CI [0.99,1.14]), but this varied significantly by antibody serostatus. The RRR was 1.16 (95% CI [1.04,1.29]) among seronegative patients and 0.97 (95% CI [0.88,1.07]) in seropositive patients [*p* = 0.02 for interaction (the difference in RRR between seropositive and seronegative patients)]. The summary hazard ratio (HR) for mortality comparing treatment to placebo was 0.81 (95% CI [0.64,1.03]) overall, 0.69 (95% CI [0.50,0.95]) in seronegative patients, and 0.96 (95% CI [0.66,1.39]) in seropositive patients (interaction *p* = 0.18). There was no evidence that the treatment effect on any outcome differed according to antigen level, whether overall or within serostatus subgroups. In regards to the composite safety outcome, the overall summary HR comparing treatment group to placebo was 0.89 (95% CI [0.66,1.21]; *Q* = 3.47 [*p* = 0.63], *I*^2^ = 0.0%), and it was 0.83 (95% CI [0.70,0.99]) and 1.04 (95% CI [0.86,1.26]) in seronegative and seropositive patients, respectively. The main limitation of the methodology is that these results are limited to the analysis of the six trials in ACTIV-3/TICO and ITAC and are not intended to be a complete summary of all trials of passive immunotherapy.

**Conclusions:**

Passive immunotherapy might be a useful treatment option for hospitalized patients with COVID-19 if administered before the appearance of endogenous antibodies. Development of similar passive immunotherapy could also be especially important during the early stages of a viral pandemic, or as novel viral variants emerge.

## Author summary

**Why was this study done?:**

- At the beginning of the COVID-19 pandemic, giving anti-COVID-19 immunotherapy to hospitalized patients was not found to be beneficial when looking at the overall population.
- It was hypothesized that immunotherapy may be beneficial when given to the subgroup of hospitalized patients with COVID-19 that had not developed their own antibody response to COVID-19.
- Therefore, we looked at six studies with a total of 3,079 patients to determine if the group without their own antibodies did better with immunotherapy.

**What did the researchers do and find?:**

- Passive immunotherapy in hospitalized patients with COVID-19 may be beneficial if administered before the patient has developed their own antibodies against COVID-19.
- There was no evidence that the treatment effect on sustained recovery and composite safety outcome varied by baseline plasma antigen levels.

**What do these findings mean?:**

- These findings tell us that during the early phase of the COVID-19 pandemic, a strategy aimed at providing the patients with immunotherapy before they are able to develop their own immune response may be beneficial.
- The main limitation of this study is that it was conducted before the COVID-19 virus had evolved to later variants, such as the Omicron variant. However, the study still provides insight into treatment approaches during the early phase of an epidemic. In addition, the current analysis focuses on the six trials that were performed using similar methodology and is not intended to represent all trials on passive immunotherapy.

## Abstract 3

An analysis of the effects of passive immunotherapy in hospitalized patients with severe COVID-19 suggests there may be some benefit for patients who have not yet developed SARS-CoV-2-specific antibodies. The findings suggest that passive immunotherapies warrant consideration at the early stage of a pandemic when immunity is not widespread.

## Introduction

While passive immunotherapy with SARS-COV-2 neutralizing monoclonal antibodies reduces hospitalization in outpatients with mild-to-moderate COVID-19 [1–3], the efficacy of passive immunotherapy in hospitalized adults with COVID-19 is less clear. Some studies have indicated that exogenous administration of neutralizing antibodies may be effective in a subgroup of hospitalized patients who lack an endogenous antibody response against SARS-CoV-2, but these findings are variable among trials [4–7]. In addition, the effect of passive immunotherapy may differ depending on SARS-CoV-2 antigen or viral RNA levels [4]. Such subgroup analyses have been limited by sample size.

The RECOVERY trial demonstrated the clinical benefit of treatment with casirivimab-imdevimab only in hospitalized patients who were seronegative for SARS-CoV-2 neutralizing antibodies at baseline [8]. However, in the RECOVERY trial, the antiviral remdesivir, which had been previously shown to have a mortality benefit, was administered in only 25% of participants. Mortality in the seronegative patients in RECOVERY was higher than that seen in other studies, raising concerns about the generalizability of the RECOVERY findings. The COVID-19 Phase 2/3 Hospitalized Trial also compared casirivimab-imdevimab to placebo and found a trend for overall improvement in clinical outcomes in those who were seronegative. However, for statistical reasons the results were considered descriptive [7]. Similarly, there appeared to be a benefit in the bamlanivimab trial in patients that lacked an endogenous neutralizing antibody response to SARS-CoV2. However, the limited sample size of the study did not allow firm conclusions [4].

The Therapeutics for Inpatients with COVID-19 (TICO) trials platform tested different passive immunotherapy strategies using similar protocols. These trials included several neutralizing monoclonal antibodies and a designed ankyrin repeat protein (DARPin) that binds the SARS-CoV-2 spike protein. The Inpatient Treatment with Anti-Coronavirus Immunoglobulin (ITAC) trial of hyperimmune intravenous immunoglobulin (hIVIG), a form of passive immunotherapy with administration of standardized neutralizing titers derived from patients that recovered from COVID-19 was also performed similarly. In contrast to RECOVERY, the TICO and ITAC trials were conducted in the setting of high rates of baseline remdesivir use (59% overall, Table 1 for individual trials). Though the TICO trials and the ITAC hIVIG trial showed no benefit for the primary outcome, several of the trials demonstrated possible benefit in the patients who did not have endogenous neutralizing antibodies at the time of enrollment [4]. However, none of the TICO trials were individually powered to detect differences in the treatment effect across subgroups, such as the presence or absence of SARS-CoV-2 endogenous antibody or the level viral RNA.

**Table 1:** Baseline characteristics of patients enrolled in the included randomized controlled trials (RCTs).

	bamlanivimab(9)	sotrovimab(5)	amubarvimab/ romlusevimab(5)	tixagevimab-cilgavimab(10)	ensovibep(11)	hIVIG(12)
No	308	261	256	1,302	414	538
*Dates of Enrollment*	Aug. 5, 2020–Oct. 13, 2020	Dec. 16, 2020–Mar. 01, 2021	Dec. 18, 2020–Mar. 01, 2021	Feb. 10, 2021- Sep. 30, 2021	Jun. 11, 2021–Nov. 15, 2021	Oct. 08, 2020–Feb. 10, 2021
*Placebo group* No (%)	149 (48.4)	84 (32.2)	85 (33.2)	613 (47.1)	174 (42.0)	264 (49.1)
*Female* No (%)	135 (43.8)	107 (41.0)	110 (43.0)	541 (41.6)	177 (42.8)	227 (42.2)
*Age* (yr) Median [IQR]	61	60	60.5	54	57	60
	[49.8–71.0]	[50.0–72.0]	[49.0–71.0]	[44.0–66.0]	[46.0–68.0]	[50.0–70.8]
*Race* No (%)						
White	178(56.8)	159 (60.9)	162 (63.3)	741 (56.0)	225 (59.3)	337 (62.6)
Black	66 (21.4)	61 (23.4)	46 (18.0)	329 (25.3)	105 (25.4)	86 (16.0)
Asian	13 (4.2)	12 (4.6)	16 (6.2)	53 (4.1)	27 (6.5)	37 (6.0)
Hispanic	73 (23.7)	33 (14.9)	52 (20.3)	240 (10.4)	70 (16.9)	89 (16.5)
Other	15 (4.9)	11 (4.2)	11 (4.3)	46 (3.5)	16 (3.9)	10(1.9)
*Symptom duration* (days) Median (IQR)	7 (5–9)	8 (5–9)	8 (5–9)	8 (6–10)	8 (5–9)	8 (6–10)
*Baseline Pulmonary ordinal scale category* No (%)						
Not receiving supplemental oxygen	86 (27.9)	88 (33.7)	81 (31.6)	303 (23.3)	79 (19.1)	135 (25.1)
Conventional supplemental oxygen <4 L/min	112 (36.4)	115 (44.1)	107 (41.8)	467 (35.9)	122 (29.5)	192 (35.7)
Conventional supplemental oxygen ≥4 L/min	63 (20.5)	58 (22.2)	68 (26.6)	388 (29.8)	130 (31.4)	154 (28.6)
High-flow nasal cannula or noninvasive ventilation	47 (15.3)	0 (0.0)	0 (0.0)	144 (11.1)	83 (20.0)	57 (10.6)
*BMI* (Kg/m^2^)	30.2	30.8	30.3	30.7	29.9	30.1
Median (IQR)	(25.9–35.9)	(26.6–37.1)	(26.6–35.5)	(26.5–36.5)	(25.7–34.9)	(26.1–35.0)
*Coexisting illness* No (%)						
Any	206 (66.9)	190 (72.8)	188 (73.4)	780 (59.9)	234 (56.5)	322 (59.9)
Hypertension	156 (50.6)	147 (56.3)	149 (58.3)	542 (41.6)	165 (39.9)	231 (42.9)
Diabetes	89 (28.9)	101 (38.7)	87 (34.0)	339 (26.0)	99 (23.9)	153 (28.4)
Renal disease	32 (10.4)	38 (14.6)	19 (7.4)	117 (9.0)	41 (9.9)	39 (7.2)
Asthma	29 (9.4)	25 (9.6)	31 (12.1)	128 (9.8)	39 (9.4)	54 (10.0)
COPD	18 (5.4)	25 (9.6)	18 (7.0)	82 (6.3)	25 (6.0)	39 (7.2)
Heart failure	17 (5.5)	22 (8.4)	28 (10.9)	56 (4.3)	22 (5.3)	30 (5.6)
Compromised immune Function	36 (11.7)	53 (20.3)	51 (19.9)	241 (18.5)	52 (12.6)	61 (11.3)
*Remdesivir prior to randomization* No (%)	123 (39.9)	168 (64.4)	161 (62.9)	820 (63.0)	282 (68.1)	276 (51.3)
*Concomitant meds* No (%)						
Corticosteroids	157 (51.0)	166 (63.6)	158 (61.7)	955 (73.3)	302 (72.9)	315 (58.6)
Antiplatelet/anticoagulant	202 (65.6)	225 (86.2)	216 (84.4)	1,065 (81.8)	313 (75.6)	412 (76.6)
Immune modulators	6 (1.9)	6 (2.3)	3 (1.2)	109 (8.4)	36 (8.7)	8 (1.5)
*SARS-COV-2 vaccine*						
Any No (%)	12 (3.9)	22 (8.4)	21 (8.2)	364 (28.0)	128 (31.2)	10 (1.9)
*Quanterix Antigen* (pq/mL)	1,015	1,450	1,370	1,656	1,314	1,368
Median (IQR)	(140–3,591)	(340–3,940)	(226–4,512)	(277–5,071)	(167–4,991]	(206–4,334)
*GenScript Anti-spike*						
Positive No (%)	155 (50.3)	106 (40.6)	109 (42.6)	695 (53.4)	246 (59.4)	261 (48.5)

Here, we present the results of a meta-analysis of six double-blind, randomized, placebo-controlled trials of passive immunotherapy in adults hospitalized with COVID-19 to determine whether clinical outcomes were improved in patients receiving passive immunotherapy, and whether these outcomes differed by baseline endogenous antibody serostatus and SARS-CoV-2 antigen level.

## Methods

### Study design

The methods and results of the TICO trials of bamlanivimab (Ly-CoV555) [9], sotrovimab [5], amubarvimab/romlusevimab (BRII-196/BRII-198) [5], tixagevimab-cilgavimab [10], and ensovibep [11], as well as the ITAC trial of hIVIG [12] have been previously reported. For all trials, patients were eligible if they were 18 years or older, had a positive test for SARS-CoV-2 infection, and required acute hospitalization for COVID-19 without need for invasive mechanical ventilation or end-organ damage. Detailed inclusion and exclusion criteria for each trial are presented in their respective publications. Participants missing baseline neutralizing antibody and antigen measurements were excluded from the present study (4% of participants across TICO and ITAC).

### Primary outcome

In the TICO trials, patients were followed for 90 days after randomization for the primary outcome of sustained recovery. Sustained recovery was defined as being discharged to home or the same level of care the patient required prior to COVID-19 diagnosis (e.g., long-term care hospital) and remaining there for 14 consecutive days. Patients in ITAC were followed for 28 days after randomization; the primary outcome was a 7-category ordinal endpoint measured at day 7 that considered pulmonary status and extrapulmonary complications. The 7 categories ranged from return to usual activities with no more than minimal symptoms due to COVID-19 to death and were based on similar outcomes in earlier influenza and COVID-19 studies. A key secondary outcome in the ITAC trial was time to discharge or the most favorable category on this ordinal scale [12]. In the present study, this secondary outcome was treated as the “time to sustained recovery” for patients in the ITAC trial [12].

### Secondary outcomes

Key secondary outcomes in the TICO trials included 90-day mortality and a composite safety outcome of grade 3 and 4 adverse events, organ failure or serious co-infection, serious adverse events, or death. In the TICO trials, grade 3 and 4 adverse events were measured through day 28, whereas mortality was assessed through day 90. Thus, we also compared 90-day mortality and composite safety outcome measured through day 28 across treatment groups in this analysis. In the ITAC trial, grade 3 and 4 adverse events were measured only through day 7 and mortality through day 28. Due to the difference in follow-up times, participants in ITAC who did not experience the composite safety endpoint by day 7 were censored for this outcome, and those who did not experience mortality by day 28 were censored at day 28 for the mortality outcome. Additionally, due to TICO being a platform study, several of the included trials shared concurrent placebo groups. Specifically, when a participant was eligible for more than one trial on the platform, they were first randomized to a trial and then randomized to the active agent or its respective matched placebo. Placebo participants who were eligible for more than one trial at the time of enrollment were subsequently included in the primary analyses of each of these trials. In the present study, a trial was defined as including the patients assigned to the relevant active treatment in addition to patients assigned to their matched placebos, but not the concurrent shared placebo participants from other trials. Therefore, each participant was assigned to only one trial, and as a result no participant assigned to placebo contributed more than once to the present study [13].

### Baseline antibody and antigen measurements

In each of the six included trials, blood samples were collected on the day of randomization prior to administration of the study intervention. SARS-CoV-2 plasma viral nucleocapsid protein levels were measured using a single-molecule immune bead assay (Quanterix, Billera, MA, USA) and a plasma surrogate neutralizing antibody assay specific for antibodies that disrupt the SARS-CoV-2 receptor binding domain (RBD)-ACE2 interaction (GenScript, Piscataway, NJ, USA).

### Statistical methods

Baseline demographics were compared across trials, with continuous variables summarized by their means and standard deviations and categorical variables summarized by frequency and proportions. A two-step individual participant data meta-analysis approach was adopted, where patient-level outcomes were first analyzed in each trial separately. The trial-level results were then pooled in a fixed (common) effect meta-analysis [14]. These results were then compared to those from one-stage models fit directly on the pooled individual participant data.

Between-trial heterogeneity was assessed visually in forest plots in conjunction with the Cochran *Q*-test for heterogeneity and *I*^2^ statistic.

Within each trial, Fine-Gray models were used to compare time to sustained recovery across treatment groups while accounting for the competing risk of death. Cox proportional hazards models were used to compare time to the composite safety outcome and time to death across treatment groups. An interaction between treatment assignment and baseline serostatus was tested to assess whether the treatment effect differed based on whether the participant tested positive for SARS-CoV-2 neutralizing antibodies at baseline. We then conducted separate analyses for the baseline seropositive and seronegative groups testing whether the effect of treatment on the rates of sustained recovery and composite safety events differed across levels of continuous antigen measurements. These differences were assessed within each trial using an interaction between treatment assignment and transformed antigen values. Antigen measurements were first log_2_ transformed. Natural cubic splines with internal knots at the 33rd and 66th percentiles of the log_2_ transformed antigen measurements were then used to allow for non-linear relationships between log_2_ transformed antigen and the relative rates of outcomes as well as the treatment effects; the boundary knots were set to coincide with the overall range of antigen measurements.

The resulting interaction coefficients from each of the above analyses were then pooled in a multivariate meta-analysis model along with the main effect of treatment to allow for the subsequent estimation of covariate-specific treatment effects. A fixed treatment effect was assumed in each analysis due to the small number of studies, similarity in the treatments assessed (all small molecules for use in passive immunotherapy) and similarity in the trial designs; the five TICO trials included were conducted under the same protocol.

Three sets of sensitivity analyses were conducted. The first removed the ensovibep (DARPin) and ITAC (hIVIG) trials to examine the results when only including trials assessing monoclonal antibody treatments. The second removed the tixagevimab-cilgavimab trial, by far the largest trial, to assess the degree to which the overall conclusions were driven by the result of this trial. The third assessed the risk of aggregation bias due to including the main effects of treatment in the multivariate meta-analysis models. Including the main effects can introduce between-study information into the estimation of the interaction coefficients if the association between an individual-level characteristic (e.g., baseline serostatus) and the treatment effect within trials differs from the association between the aggregated trial-level characteristic (e.g., proportion seropositive at baseline) and the trial-level treatment effects that would be observed in a meta-regression [15,16]. To assess this, separate models were fit pooling only the interaction terms, thus using only within-trial information; the point estimates for the interaction coefficients and the p-values for the test of the interaction were then compared between approaches. For the interaction between treatment and continuous log_2_ transformed antigen levels, estimated sustained recovery rate ratio (RRR) curves were compared when pooling the main effect of treatment jointly with the interaction coefficients to when the main effect was assumed to be independent from the interaction terms. This independence assumption avoids incorporating between-study information into the estimated interaction coefficients [15].

All analyses were conducted using R version 4.1.2 [17]. The “cmprsk” [18] and “survival” [19] packages were used to fit the Fine-Gray and Cox proportional hazards models, respectively. The “mixmeta” package was used to fit all meta-analysis models [20]. No correction for multiple comparisons was performed, and *p*-values less than 0.05 were considered statistically significant for all analyses.

### Ethical considerations

The study was conducted according to the Declaration of Helsinki in its current version; the requirements of Good Clinical Practice (GCP) as defined in Guidelines, EU Clinical Trials Directive (2001/20/EC), and EU GCP Directive (2005/28/EC); International Council for Harmonisation of Technical Requirements for Pharmaceuticals for Human Use (ICH) Guidelines; Human Subject Protection and Data Protection Acts; the US Office for Human Research Protections (OHRP); or with the local law and regulation, whichever afforded greater protection of human subjects.

## Results

### Characteristics of the population of each included trial

The six trials included in this meta-analysis enrolled a total of 3,079 patients with baseline antibody and antigen measurements with trial size ranging between 256 in the amubarvimab/romlusevimab** **trial and 1,302 in the tixagevimab-cilgavimab trial (Table 1). Enrollment in the trials took place during the second half of 2020 and early 2021 for bamlanivimab ([9], sotrovimab [5], amubarvimab/romlusevimab [5], and hIVIG [12] and February 2021 to the second half of 2021 for tixagevimab-cilgavimab and ensovibep [11]. The duration of symptoms was between seven and eight days prior to enrollment. Compared to patients in the other trials, those in the tixagevimab-cilgavimab and ensovibep trials tended to be younger and, based on the “baseline pulmonary ordinal scale category,” had more severe disease at enrollment [10]. They were also more likely to have been treated with corticosteroids or immune modulators at baseline. Reflecting the different enrollment periods, the percentage of patients who had received at least one dose of an anti-SARS-CoV-2 vaccine was minimal for bamlanivimab, sotrovimab, amubarvimab/romlusevimab, and hIVIG trials ranging from 1.9% to 8.4%. The corresponding percentages for tixagevimab-cilgavimab and ensovibep trials were 28% and 31.2%, respectively.

At trial entry, the percentage of patients seropositive for endogenous neutralizing antibodies ranged from 40.6% to 59.4%, the highest in the ensovibep trial followed by the tixagevimab-cilgavimab trial (53.4%). The overall median plasma antigen level was 1,421 pg/mL (IQR: 231–4,568), ranging from 1,015 pg/mL (bamlanivimab trial) to 1,656 pg/mL (tixagevimab-cilgavimab trial). Additional characteristics of the patients included in each trial are summarized in Table 1, and further description can be found in the original publication of each trial [5,9–12].

### Treatment effect by antibody status at trial entry

#### (a) *Sustained recovery.*

**Fig 1:**
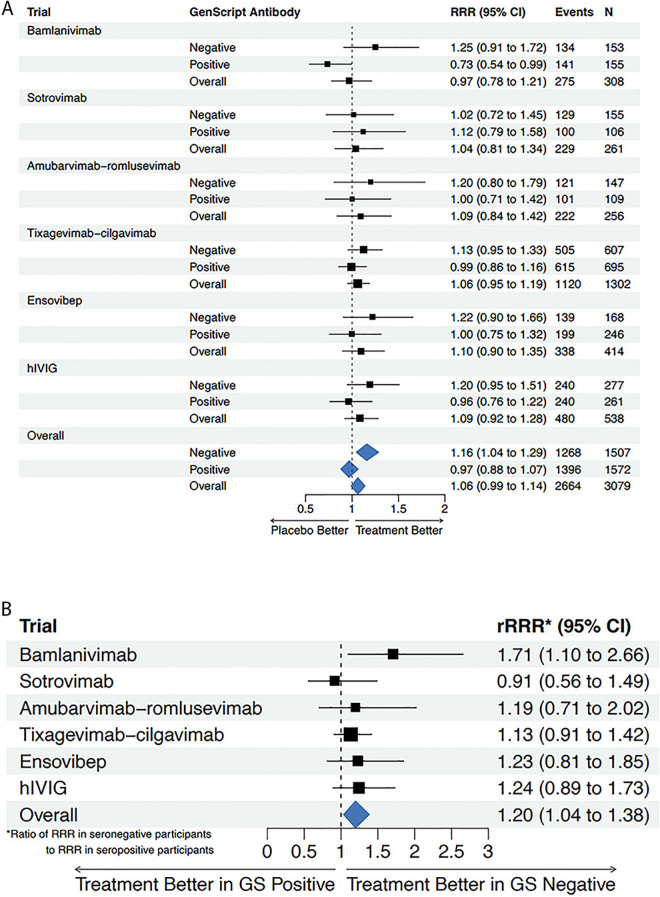
Sustained recovery by baseline SARS-CoV-2 neutralizing antibody status. **(a)** Forest plot of recovery rate ratio (RRR) comparing treatment arm versus matched placebo by neutralizing antibody status at study entry. **(b)** Forest plot of the relative recovery rate ratio (rRRR) comparing the estimated treatment effect in neutralizing antibody seropositive versus seronegative patients at study entry.

By the end of the 90-day follow-up period (28 days for hIVIG), sustained recovery was achieved by 1,494 of 1,710 patients (87%) assigned to receive active treatment, and by 1,170 of 1,369 patients (85%) assigned to placebo. Among participants seronegative at baseline for neutralizing antibodies, this was 702 of 815 (86%) assigned to active treatment, and 566 of 692 (82%) assigned to placebo; among seropositive participants, 792 of 895 (88%) assigned to active treatment and 604 of 677 (89%) assigned to placebo achieved sustained recovery. The forest plot in Fig 1A displays the treatment effect, as measured by the sustained RRR for treatment versus placebo. The overall summary estimate of the RRR was 1.06 (95% CI [0.99,1.14]). There was little evidence of between-trial heterogeneity in the treatment effect on the rate of sustained recovery (*Q* = 0.89 [*p* = 0.97]; *I*^2^ = 0.00). Study-specific cumulative incidence curves for sustained recovery are shown in S1 Fig. The treatment effect varied significantly by baseline neutralizing antibody serostatus with greater benefit among seronegative patients; the summary estimate of the RRR was 1.16 (95% CI [1.04,1.29]) among seronegative patients and 0.97 (95% CI [0.88,1.07]) in seropositive patients (Fig 1A). The estimated summary RRR among seronegative patients was 20% greater than among seropositive patients (95% CI [4%, 38%]; pooled interaction *p* = 0.02) (Fig 1B). Similar to the overall treatment effect, there was little evidence of heterogeneity in the difference in the RRRs between baseline seronegative and seropositive participants across the trials (*Q* = 3.95 [*p* = 0.56], *I*^2^ = 0.0%).

#### (b) *Mortality and composite safety outcome.*

**Fig 2:**
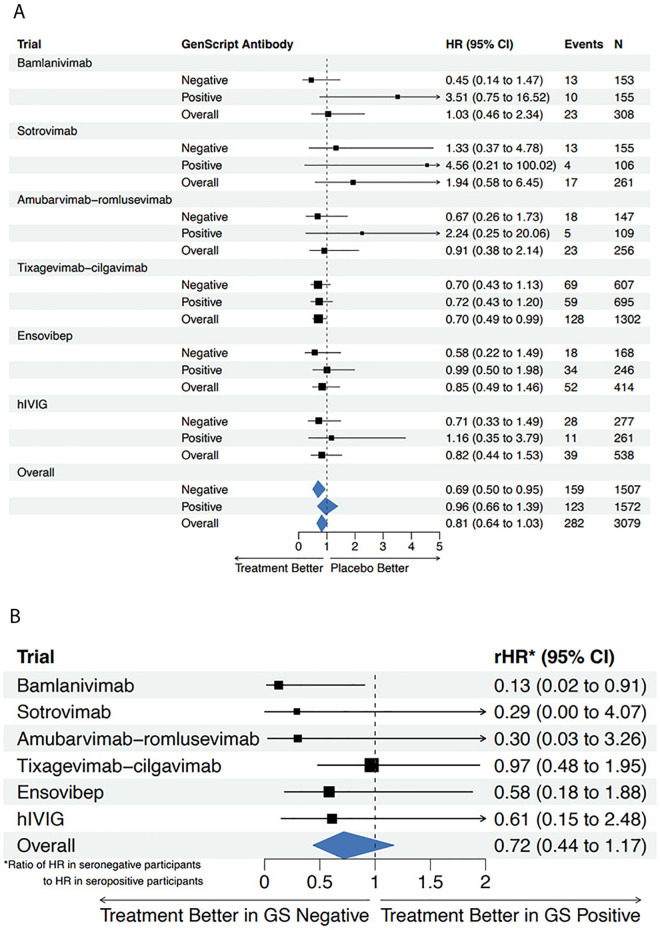
Mortality by baseline SARS-CoV-2 neutralizing antibody status. **(a)** Forest plot of hazard ratio (HR) for mortality comparing treatment arm versus matched placebo by neutralizing antibody status at study entry. **(b)** Forest plot of the relative hazard ratio (rHR) comparing the estimated treatment effect in neutralizing antibody seropositive versus seronegative patients at study entry.

There were 144 deaths among 1,710 patients (8.4%) randomized to passive immunotherapy (seronegative: 73/815 [9.0%]; seropositive: 71/895 [7.9%]) and 138 deaths among 1,369 patients (10.1%) randomized to placebo (seronegative: 86/692 [12.4%]; seropositive: 52/677 [7.7%]). The overall summary (fixed effect pooled estimate) of the hazard ratio (HR) for mortality comparing treatment versus placebo was 0.81 (95% CI [0.64,1.03]). As observed with sustained recovery, there was little evidence of between-trial heterogeneity in the treatment effect (*Q* = 3.13, *p* = 0.68, *I*^2^ = 0.0%). Study-specific cumulative incidence curves for mortality are shown in S2 Fig. Consistent with the findings regarding the heterogeneity of the treatment effect on sustained recovery by baseline neutralizing antibodies serostatus, the estimated treatment effect on mortality was larger in seronegative than in seropositive patients, though this difference was not statistically significant (pooled interaction *p* = 0.18; *Q* = 4.54 [*p* = 0.47], I^2^ = 0.0%). The summary HR was 0.69 (95% CI [0.50,0.95]) in seronegative patients and 0.96 (95% CI [0.66,1.39]) in seropositive patients (Fig 2A). The HR in seronegative patients was 28% lower than the HR among seropositive patients (95% CI [-56%, 17%]; pooled interaction *p* = 0.18) (Fig 2B).

Similar results were found when the day 28 composite safety outcome was considered (S3 Fig). The overall summary HR comparing treatment group to placebo was 0.89 (95% CI [0.66,1.21]; *Q* = 3.47 [*p* = 0.63], *I*^2^ = 0.0%), and it was 0.83 (95% CI [0.70,0.99]) and 1.04 (95% CI [0.86,1.26) in seronegative and seropositive patients, respectively. Study-specific cumulative incidence curves for composite safety are shown in S4 Fig. The summary HR was 20% lower among seronegative patients compared to seropositive patients (95% CI [-38%, 3%]; pooled interaction *p* = 0.09; *Q* = 3.99 [*p* = 0.55], *I*^2^ = 0.0%), though this was again not statistically significant. Similar one-stage models fit directly on the individual participant data for each of the above analyses yielded similar results.

### Treatment effect by baseline plasma antigen levels

#### (a) *Sustained recovery.*

**Fig 3:**
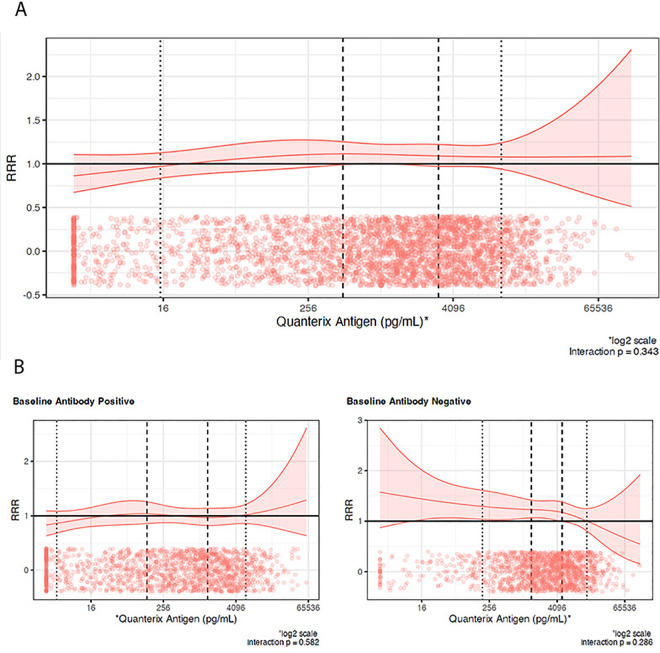
Pooled recovery rate ratio (RRR) for sustained recovery comparing treatment arm vs. matched placebo by baseline plasma antigen level. **(a)** Overall Study, **(b)** By neutralizing antibody status at study entry. The points show the observed distribution of baseline antigen measurements in patients. The dashed lines represent the 33rd and 66th percentiles of antigen measurements across trials in the respective patient group (overall, seropositive, seronegative); these correspond to the locations of the internal knots for the restricted cubic splines. The dotted lines represent the 10th and 90th percentiles of antigen measurements in the respective group.

The estimated summary RRR for sustained recovery, with a 95% confidence band, is plotted against baseline plasma antigen level in Fig 3A; the corresponding trial-specific curves are shown in S5 Fig. Overall, there was no evidence that the treatment effect on sustained recovery varied by baseline plasma antigen levels (pooled interaction *p* = 0.34) with the 95% confidence band of the estimated RRR curve including 1 across all levels of baseline antigen levels.

As the treatment effect on sustained recovery differed by baseline neutralizing antibodies serostatus, the association of the treatment effect on sustained recovery by baseline plasma antigen level was also considered separately by baseline neutralizing antibody serostatus. The estimated curves of RRR by antigen levels for the seropositive and seronegative subgroups of patients are shown, for each trial, in S6 and S7 Figs, respectively. The corresponding summary RRR curves, pooled across all trials, are shown in Fig 3B. In seropositive patients, there was no indication of any treatment effect on sustained recovery across all baseline antigen levels. The 95% confidence band of the estimated RRR curve included the value of 1 with the summary RRR being roughly 1 across all levels of baseline plasma antigen levels; the *p*-value for the interaction was equal to 0.58.

Similarly, in the seronegative sub-group, there was no evidence that treatment effect differed according to antigen level (*p*-value for interaction = 0.29; Fig 3B). Excluding the ensovibep and hIVIG trials (S8 Fig) or the tixagevimab-cilgavimab trial (S9 Fig) resulted in similar results as above for both the neutralizing antibody seropositive and seronegative groups. Using only the within-trial information when estimating the pooled estimated RRR curves also led to similar results within both the seropositive and seronegative groups (S10 Fig). The results were also similar when excluding patients with high-flow nasal cannula or noninvasive ventilation for both the seropositive and seronegative groups (S11 Fig). It should be noted though that none of the patients enrolled in the sotrovimab and the amubarvimab/romlusevimab trials were in this category (Table 1).

#### (b) *Composite safety outcome.*

For the day 28 composite safety outcome, estimated HR curves by baseline antigen levels were heterogenous across trials both overall (S12 Fig) and when analyzing the neutralizing antibody seropositive and seronegative groups separately (S13 and S14 Figs). The estimated pooled HR was near 1 across all baseline antigen levels overall (S15 Fig) as well as in the neutralizing antibody seronegative and seropositive subgroups. However, in the seropositive subgroup there was a trend towards higher HRs at lower baseline antigen levels and lower HRs at higher baseline antigen levels (S16 Fig), though this was not statistically significant (pooled interaction *p* = 0.07). Sensitivity analyses excluding the ensovibep and hIVIG trials (S17 Fig) or excluding the tixagevimab-cilgavimab trial (S18 Fig) led to similar results to the main analysis, as did the sensitivity analysis using only the within-trial information to estimate the pooled HR curves (S19 Fig). When patients with high flow nasal cannula or noninvasive ventilation at baseline were excluded (S20 Fig), the results remained unchanged although the *p*-value for differentiated effects of treatment by antigen levels was now below 0.05 for the neutralizing antibody seropositive group.

## Discussion

This study is an individual patient data meta-analysis of six randomized placebo-controlled trials of passive immunotherapies involving 3,079 patients hospitalized with documented SARS-CoV-2 infection and COVID-19 symptoms for ≤12 days. Five out of six of the trials were conducted on the ACTIV-3/TICO platform, with identical definitions of the primary endpoint and harmonized data collection. In addition, the hIVIG trial, ITAC, was also included because it was conducted contemporaneously by the same study group using very similar study procedures, albeit with a shorter follow-up period. As such, we did not include trials with convalescent plasma. A major objective of the study was to compare the effect of passive immunotherapy across patients’ characteristics with the aim of identifying subgroups of patients that may benefit most from the treatment. We found that overall, there was not a significant association between receiving passive immunotherapy and the rate of sustained recovery. There was a trend, though not statistically significant, towards lower mortality in the treatment group (19% reduction; HR: 0.81, 95% CI: 0.64,1.03), and the study may have been underpowered for this outcome. The effect of passive immunotherapy, compared to placebo, varied according to anti-SARS-CoV-2 serostatus at enrollment with statistically significant benefit seen only among patients who were antibody negative, but not in those who were antibody positive.

Although the apparent heterogeneity in the treatment effect on mortality by antibody status was not statistically significant, it is consistent with findings from other trials. In the RECOVERY trial, casirivimab−imdevimab was associated with a significant reduction of 28-day mortality (21%) in seronegative patients with no evidence of effect among seropositive patients [8]. The RECOVERY trial included a total of 3,153 seronegative patients with 848 deaths (27%). The lack of compelling evidence for the dependence of the mortality effect of passive immunotherapy on serostatus in our study may be due to insufficient power with a total of 1,507 seronegative patients with 123 deaths (11%). Another important difference between this study and RECOVERY is that the antiviral remdesivir was included in TICO protocol. Only a small percentage, about 25%, of RECOVERY patients had received remdesivir and the overall mortality was substantially higher. Therefore, the current study demonstrates the effect of passive immunotherapy in seronegative patients even in the presence of standard immunosuppressive and antiviral therapy. It may also be that the role of antibody serostatus as a modifier of the treatment effect on mortality is dependent on the specific passive immunotherapy product, and the largest trial in this meta-analysis (comparing tixagevimab-cilgavimab versus placebo) resulted in similar efficacy for mortality regardless of serostatus [10].

In the current study, there was evidence for heterogeneity in the passive immunotherapy effect by baseline antibody status on sustained recovery and a marginally significant trend towards a difference for the composite safety endpoint. For both events, a significant passive immunotherapy benefit was found for seronegative patients but not for seropositive patients. Given the larger number of events in these outcomes, it is more likely that the lack of clear evidence of heterogeneity of the treatment effect on mortality by serostatus is due to low power.

There are limitations to consider for interpretation of these data. First, while the number of patients was sufficient in this study for assessing the effect of the prespecified patient characteristics (antibody serostatus and plasma antigen level) on the treatment effect on sustained recovery, this may have been insufficient for mortality (Fig 2B). Specifically, the lack of statistically significant interactions does not imply the absence of treatment effect heterogeneity. Second, the majority (82%) of patients included in this analysis were not vaccinated, so it is unclear whether our findings regarding efficacy of passive immunotherapy and, in particular, whether the relationship of the benefit of passive immunotherapy to antibody serostatus will be relevant to a vaccinated and COVID-exposed population. Third, all six of the trials included in this meta-analysis were fully enrolled before the appearance of the omicron variant, so the applicability of our findings to patients infected with omicron and later variants is unknown since the antibodies are less effective against different variants. Importantly, however, these findings give insight into results that might be anticipated using passive immunotherapy for hospitalized patients during the early stages of a future pandemic. Hyperimmune plasma as represented by the ITAC trial may be a readily available product that could be deployed as new viral variants emerge.

Other strengths of this meta-analysis include the availability and use of individual patient data from randomized double-blind placebo-controlled trials conducted under very similar protocols (with five using largely the same protocol and data collection procedures). This made it possible to compare common outcomes of subgroups of patients within trials and then combine these results across trials, thus reducing the risk of aggregation bias that can arise from meta-regression of trial-level characteristics.

In conclusion, this meta-analysis suggests that, for patients hospitalized with COVID-19, passive immunotherapy might be a useful treatment option if administered before the appearance of endogenous antibodies. These data that were obtained in the early stages of the COVID-19 pandemic may be applicable to future pandemics or as new variants of SARS-CoV-2 emerge that are resistant to currently available therapeutics. We have demonstrated that passive immunotherapy in antibody-negative patients may expedite recovery and save lives early in a pandemic before widespread population-level immunity is achieved.

## Supporting Information

S1 Fig Study-specific cumulative incidence curves for sustained recovery overall and by baseline neutralizing antibody serostatus. (PDF)

S2 Fig Study-specific cumulative incidence curves for mortality overall and by baseline neutralizing antibody serostatus. (PDF)

S3 Fig Day-28 Composite safety outcome by baseline SARS-CoV-2 neutralizing antibody status: forest plot of hazard ratios (HR) comparing treatment arm versus matched placebo by neutralizing antibody status at study entry (*p*-value for pooled test of interaction = 0.087). (PDF)

S4 Fig Study-specific cumulative incidence curves for composite safety overall and by baseline neutralizing antibody serostatus. (PDF)

S5 Fig Estimated recovery rate ratio (RRR) for sustained recovery comparing treatment arm versus matched placebo by baseline plasma antigen levels (log2 scale) within each trial. The points show the observed distribution of baseline antigen measurements. The dashed lines represent the overall 33rd and 66th percentiles of antigen measurements across trials; these correspond to the locations of the internal knots for the restricted cubic splines.
(PDF)

S6 Fig Recovery rate ratio (RRR) for sustained recovery comparing treatment arm versus matched placebo by baseline plasma antigen levels (log2 scale) within each trial among patients neutralizing antibody positive at study entry. The points show the observed distribution of baseline antigen measurements. The dashed lines represent the 33rd and 66th percentiles of antigen measurements across trials in the seropositive subgroup; these correspond to the locations of the internal knots for the restricted cubic splines.
(PDF)

S7 Fig Recovery rate ratio (RRR) for sustained recovery comparing treatment arm versus matched placebo by baseline plasma antigen levels (log2 scale) within each trial among patients neutralizing antibody negative at study entry. The points show the observed distribution of baseline antigen measurements in patients. The dashed lines represent the 33rd and 66th percentiles of antigen measurements across trials in the seronegative subgroup; these correspond to the locations of the internal knots for the restricted cubic splines.
(PDF)

S8 Fig Pooled recovery rate ratio (RRR) for sustained recovery comparing treatment arm versus matched placebo by baseline plasma antigen level for: (a) patients neutralizing antibody positive, and (b) patients neutralizing antibody negative at study entry when excluding the ensovibep and hIVIG trials. The points show the observed distribution of baseline antigen measurements in patients. The dashed lines represent the 33rd and 66th percentiles of antigen measurements across trials for the respective patient group (seropositive, seronegative); these correspond to the locations of the internal knots for the restricted cubic splines. The dotted lines represent the 10th and 90th percentiles of antigen measurements in the respective group.
(PDF)

S9 Fig Pooled recovery rate ratio (RRR) for sustained recovery comparing treatment arm versus matched placebo by baseline plasma antigen level for: (a) patients neutralizing antibody positive, and (b) patients neutralizing antibody negative at study entry when excluding the tixagevimab-cilgavimab trial. The points show the observed distribution of baseline antigen measurements in patients. The dashed lines represent the 33rd and 66th percentiles of antigen measurements across trials for the respective patient group (seropositive, seronegative); these correspond to the locations of the internal knots for the restricted cubic splines. The dotted lines represent the 10th and 90th percentiles of antigen measurements in the respective group.
(PDF)

S10 Fig Pooled recovery rate ratio (RRR) for sustained recovery comparing treatment arm versus matched placebo by baseline plasma antigen level for: (a) patients neutralizing antibody positive, and (b) patients neutralizing antibody negative at study entry when using only within-trial information to estimate the interaction coefficients (blue line). The points show the observed distribution of baseline antigen measurements in patients. The dashed lines represent the 33rd and 66th percentiles of antigen measurements across trials for the respective patient group (seropositive, seronegative); these correspond to the locations of the internal knots for the restricted cubic splines. The dotted lines represent the 10th and 90th percentiles of antigen measurements in the respective group.
(PDF)

S11 Fig Pooled recovery rate ratio (RRR) for sustained recovery comparing treatment arm versus matched placebo by baseline plasma antigen level for: (a) patients neutralizing antibody positive, and (b) patients neutralizing antibody negative at study entry when excluding patients on high flow nasal canula (HFNC) at study entry. The points show the observed distribution of baseline antigen measurements in patients. The dashed lines represent the 33rd and 66th percentiles of antigen measurements across trials for the respective patient group (seropositive, seronegative); these correspond to the locations of the internal knots for the restricted cubic splines. The dotted lines represent the 10th and 90th percentiles of antigen measurements in the respective group.
(PDF)

S12 Fig Estimated hazard ratio (HR) for the composite safety outcome comparing treatment arm versus matched placebo by baseline plasma antigen levels (log2 scale) within each trial. The points show the observed distribution of baseline antigen measurements. The dashed lines represent the overall 33rd and 66th percentiles of antigen measurements across trials; these correspond to the locations of the internal knots for the restricted cubic splines.
(PDF)

S13 Fig Estimated hazard ratio (HR) for the composite safety outcome comparing treatment arm versus matched placebo by baseline plasma antigen levels (log2 scale) within each trial among patients neutralizing antibody positive at study entry. The points show the observed distribution of baseline antigen measurements. The dashed lines represent the 33rd and 66th percentiles of antigen measurements across trials in the seropositive subgroup; these correspond to the locations of the internal knots for the restricted cubic splines.
(PDF)

S14 Fig Estimated hazard ratio (HR) for the composite safety outcome comparing treatment arm versus matched placebo by baseline plasma antigen levels (log2 scale) within each trial among patients neutralizing antibody negative at study entry. The points show the observed distribution of baseline antigen measurements. The dashed lines represent the 33rd and 66th percentiles of antigen measurements across trials in the seronegative subgroup; these correspond to the locations of the internal knots for the restricted cubic splines.
(PDF)

S15 Fig Pooled hazard ratio (HR) for the composite safety outcome comparing treatment arm versus matched placebo by baseline plasma antigen level for the overall trial population. The points show the observed distribution of baseline antigen measurements in patients. The dashed lines represent the 33rd and 66th percentiles of antigen measurements across trials; these correspond to the locations of the internal knots for the restricted cubic splines. The dotted lines represent the 10th and 90th percentiles of antigen measurements.
(PDF)

S16 Fig Pooled hazard ratio (HR) for the composite safety outcome comparing treatment arm versus matched placebo by baseline plasma antigen level for: (a) patients neutralizing antibody positive, and (b) patients neutralizing antibody negative at study entry. The points show the observed distribution of baseline antigen measurements in patients. The dashed lines represent the 33rd and 66th percentiles of antigen measurements across trials the respective patient group (seropositive, seronegative); these correspond to the locations of the internal knots for the restricted cubic splines. The dotted lines represent the 10th and 90th percentiles of antigen measurements in the respective group.
(PDF)

S17 Fig Pooled hazard ratio (HR) for the composite safety outcome comparing treatment arm versus matched placebo by baseline plasma antigen level for: (a) patients neutralizing antibody positive, and (b) patients neutralizing antibody negative at study entry when excluding the ensovibep and hIVIG trials. The points show the observed distribution of baseline antigen measurements in patients. The dashed lines represent the 33rd and 66th percentiles of antigen measurements across trials for the respective patient group (seropositive, seronegative); these correspond to the locations of the internal knots for the restricted cubic splines. The dotted lines represent the 10th and 90th percentiles of antigen measurements in the respective group.
(PDF)

S18 Fig Pooled hazard ratio (HR) for the composite safety outcome comparing treatment arm versus matched placebo by baseline plasma antigen level for: (a) patients neutralizing antibody positive, and (b) patients neutralizing antibody negative at study entry when excluding the tixagevimab-cilgavimab trial. The points show the observed distribution of baseline antigen measurements in patients. The dashed lines represent the 33rd and 66th percentiles of antigen measurements across trials for the respective patient group (seropositive, seronegative); these correspond to the locations of the internal knots for the restricted cubic splines. The dotted lines represent the 10th and 90th percentiles of antigen measurements in the respective group.
(PDF)

S19 Fig Pooled hazard ratio (HR) for the composite safety outcome comparing treatment arm versus matched placebo by baseline plasma antigen level for: (a) patients neutralizing antibody positive, and (b) patients neutralizing antibody negative at study entry when using only within-trial information to estimate the interaction coefficients (blue line). The points show the observed distribution of baseline antigen measurements in patients. The dashed lines represent the 33rd and 66th percentiles of antigen measurements across trials for the respective patient group (seropositive, seronegative); these correspond to the locations of the internal knots for the restricted cubic splines. The dotted lines represent the 10th and 90th percentiles of antigen measurements in the respective group.
(PDF)

S20 Fig Pooled hazard ratio (HR) for the composite safety outcome comparing treatment arm versus matched placebo by baseline plasma antigen level for: (a) patients neutralizing antibody positive, and (b) patients neutralizing antibody negative at study entry when excluding patients on high flow nasal canula (HFNC) at study entry. The points show the observed distribution of baseline antigen measurements in patients. The dashed lines represent the 33rd and 66th percentiles of antigen measurements across trials for the respective patient group (seropositive, seronegative); these correspond to the locations of the internal knots for the restricted cubic splines. The dotted lines represent the 10th and 90th percentiles of antigen measurements in the respective group.
(PDF)

## Abbreviations

DARPin: designed ankyrin repeat protein
hIVIG: hyperimmune intravenous immunoglobulin
HR: hazard ratio
ITAC: Inpatient Treatment with Anti-Coronavirus Immunoglobulin
mAb: monoclonal antibodies
RRR: recovery rate ratio
TICO: Therapeutics for Inpatients with COVID-19
