# Supplementary material for: Passive immunotherapy for adults hospitalized with COVID-19: An individual participant data meta-analysis of six randomized controlled trials
Source: PLoS Med. 2025 Jul 7;22(7):e1004616. doi: 10.1371/journal.pmed.1004616 (PMC12282900; doi:10.1371/journal.pmed.1004616)

Supplemental Figures

S1 Fig. Study specific cumulative incidence curves for sustained recovery overall and by baseline neutralizing antibody serostatus

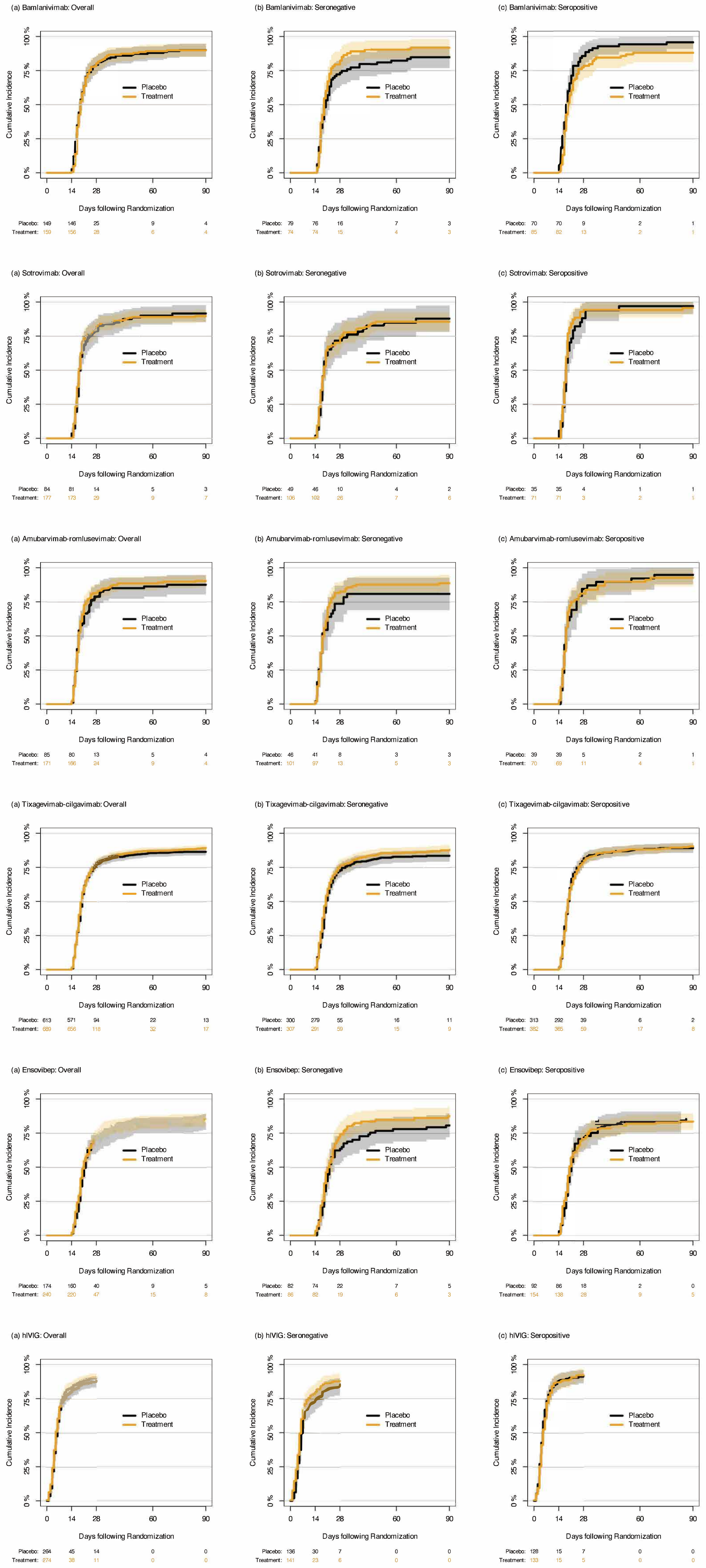

Supplement: S1 Fig — (PDF) [file pmed.1004616.s001.pdf]
