# Supplementary material for: Passive immunotherapy for adults hospitalized with COVID-19: An individual participant data meta-analysis of six randomized controlled trials
Source: PLoS Med. 2025 Jul 7;22(7):e1004616. doi: 10.1371/journal.pmed.1004616 (PMC12282900; doi:10.1371/journal.pmed.1004616)

S3 Fig. Day-28 Composite safety outcome by baseline SARS-CoV-2 neutralizing antibody status: forest plot of hazard ratios (HR) comparing treatment arm versus matched placebo by neutralizing antibody status at study entry (p-value for pooled test of interaction = 0.087).

Trial	GenScript Antibody	HR (95% CI)	Events	N
Bamlanivimab				
	Negative	1.04 (0.60 to 1.81)	50	153
	Positive	1.61 (0.84 to 3.08)	40	155
	Overall	1.23 (0.81 to 1.87)	90	308
Sotrovimab				
	Negative	0.84 (0.48 to 1.47)	54	155
	Positive	0.72 (0.32 to 1.60)	25	106
	Overall	0.80 (0.51 to 1.27)	79	261
Amubarvimab–romlusevimab				
	Negative	0.98 (0.53 to 1.80)	48	147
	Positive	1.23 (0.62 to 2.46)	37	109
	Overall	1.08 (0.69 to 1.71)	85	256
Tixagevimab–cilgavimab				
	Negative	0.82 (0.63 to 1.06)	233	607
	Positive	1.02 (0.77 to 1.35)	194	695
	Overall	0.89 (0.74 to 1.08)	427	1302
Ensovibep				
	Negative	0.90 (0.56 to 1.46)	68	168
	Positive	0.77 (0.50 to 1.20)	82	246
	Overall	0.81 (0.59 to 1.11)	150	414
hIVIG				
	Negative	0.68 (0.46 to 1.01)	103	277
	Positive	1.31 (0.81 to 2.14)	66	261
	Overall	0.89 (0.66 to 1.21)	169	538
Overall				
	Negative	0.83 (0.70 to 0.99)	556	1507
	Positive	1.04 (0.86 to 1.26)	444	1572
	Overall	0.91 (0.80 to 1.03)	1000	3079

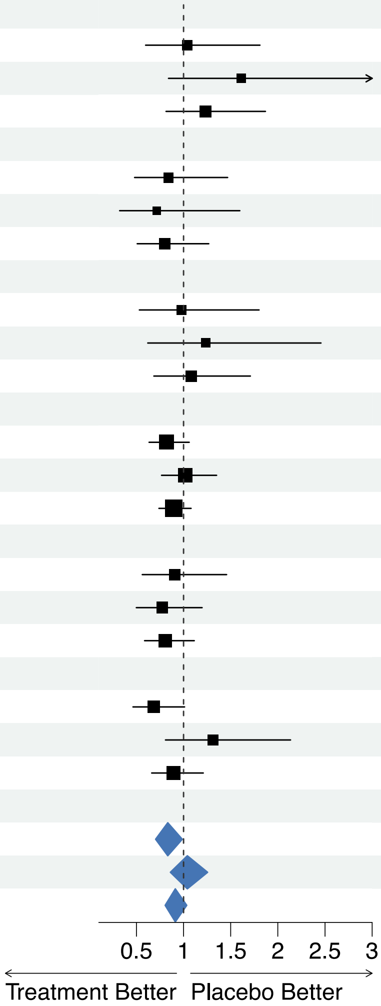

Supplement: S3 Fig — (PDF) [file pmed.1004616.s003.pdf]
