# Supplementary material for: Passive immunotherapy for adults hospitalized with COVID-19: An individual participant data meta-analysis of six randomized controlled trials
Source: PLoS Med. 2025 Jul 7;22(7):e1004616. doi: 10.1371/journal.pmed.1004616 (PMC12282900; doi:10.1371/journal.pmed.1004616)

S5 Fig. Estimated recovery rate ratio (RRR) for sustained recovery comparing treatment arm versus matched placebo by baseline plasma antigen levels (log_2 scale) within each trial. The points show the observed distribution of baseline antigen measurements. The dashed lines represent the overall 33rd and 66th percentiles of antigen measurements across trials; these correspond to the locations of the internal knots for the restricted cubic splines.

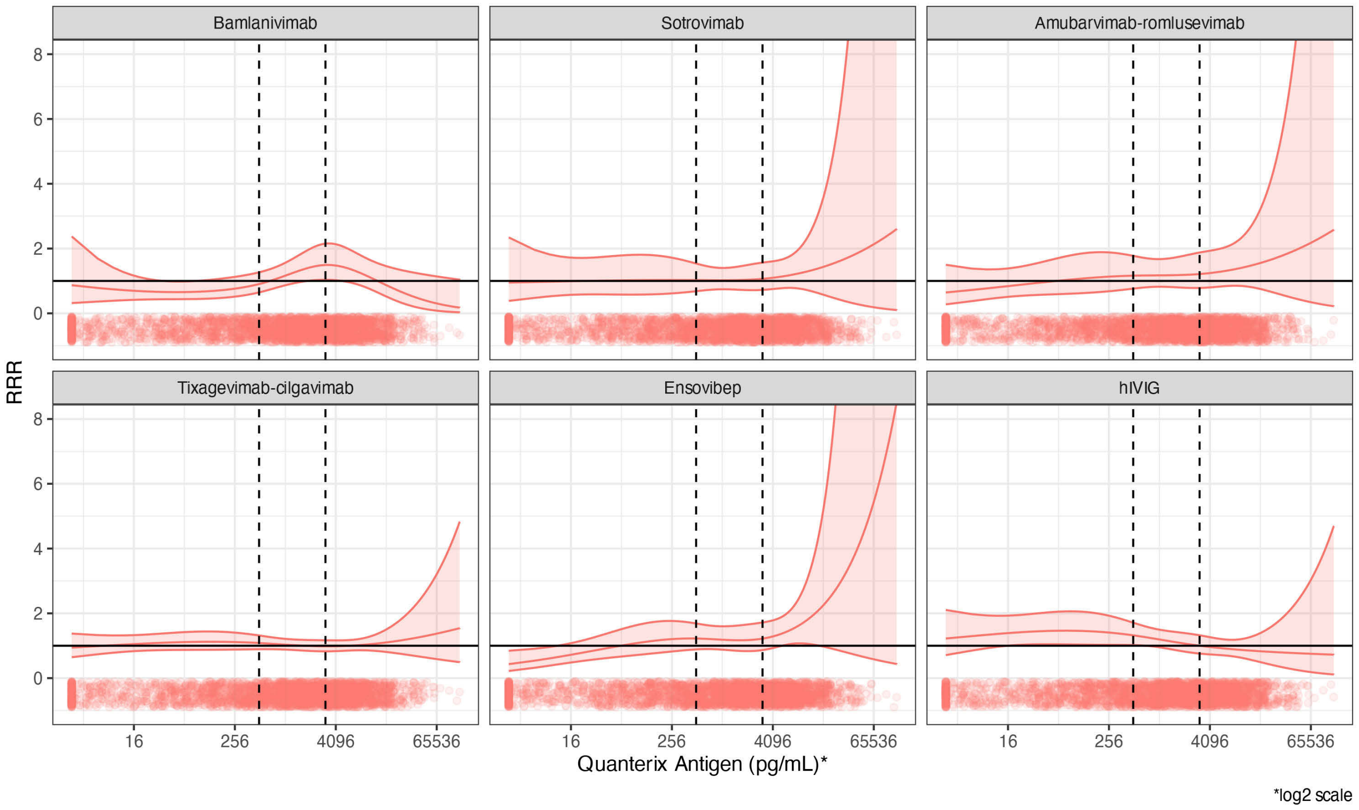

Supplement: S5 Fig — The points show the observed distribution of baseline antigen measurements. The dashed lines represent the overall 33rd and 66th percentiles of antigen measurements across trials; these correspond to the locations of the internal knots for the restricted cubic splines. (PDF) [file pmed.1004616.s005.pdf]
