# Supplementary material for: Passive immunotherapy for adults hospitalized with COVID-19: An individual participant data meta-analysis of six randomized controlled trials
Source: PLoS Med. 2025 Jul 7;22(7):e1004616. doi: 10.1371/journal.pmed.1004616 (PMC12282900; doi:10.1371/journal.pmed.1004616)

S6 Fig. Recovery rate ratio (RRR) for sustained recovery comparing treatment arm versus matched placebo by baseline plasma antigen levels ($\log_2$ scale) within each trial among patients neutralizing antibody positive at study entry. The points show the observed distribution of baseline antigen measurements. The dashed lines represent the 33rd and 66th percentiles of antigen measurements across trials in the seropositive subgroup; these correspond to the locations of the internal knots for the restricted cubic splines.

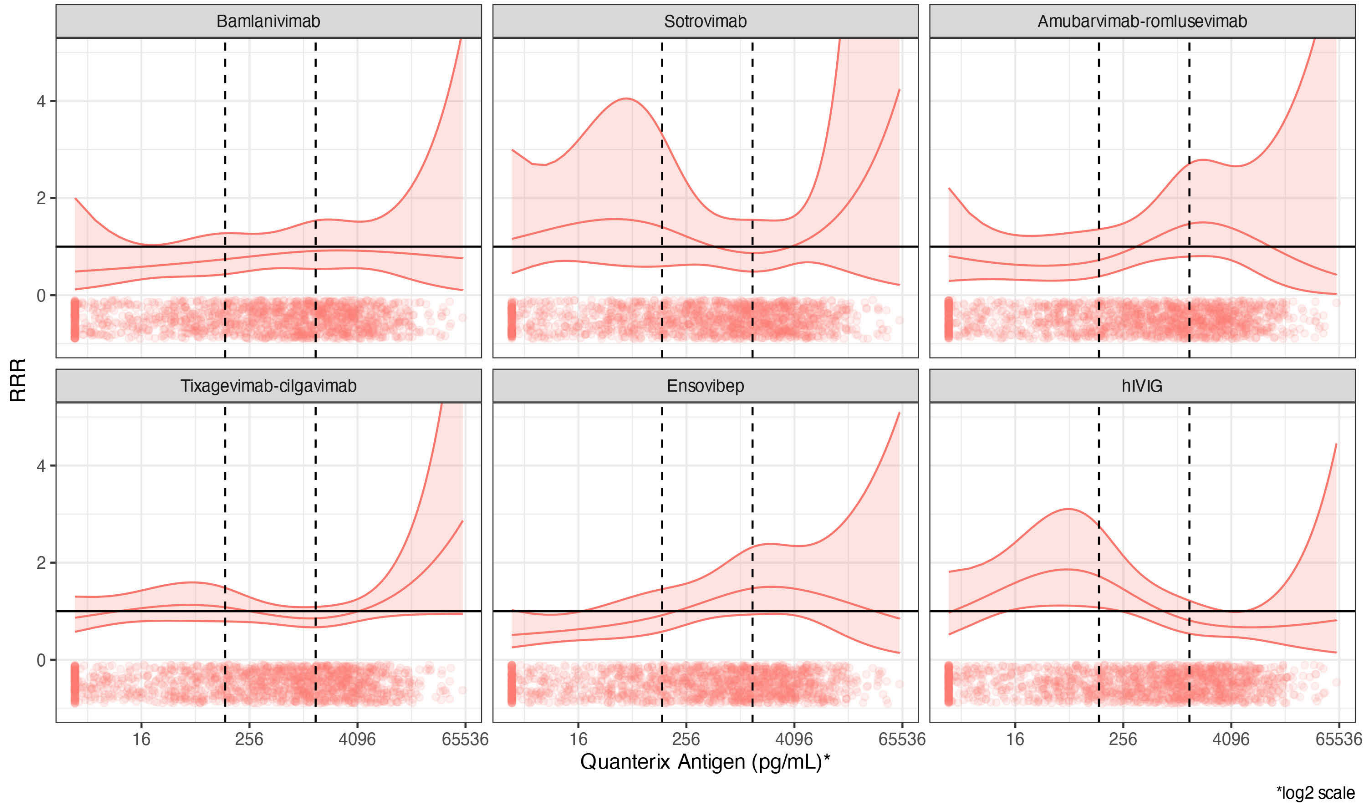

Supplement: S6 Fig — The points show the observed distribution of baseline antigen measurements. The dashed lines represent the 33rd and 66th percentiles of antigen measurements across trials in the seropositive subgroup; these correspond to the locations of the internal knots for the restricted cubic splines. (PDF) [file pmed.1004616.s006.pdf]
