# Supplementary material for: Passive immunotherapy for adults hospitalized with COVID-19: An individual participant data meta-analysis of six randomized controlled trials
Source: PLoS Med. 2025 Jul 7;22(7):e1004616. doi: 10.1371/journal.pmed.1004616 (PMC12282900; doi:10.1371/journal.pmed.1004616)

S18 Fig. Pooled hazard ratio (HR) for the composite safety outcome comparing treatment arm versus matched placebo by baseline plasma antigen level for: (a) patients neutralizing antibody positive, and (b) patients neutralizing antibody negative at study entry when excluding the tixagevimab-cilgavimab trial. The points show the observed distribution of baseline antigen measurements in patients. The dashed lines represent the 33rd and 66th percentiles of antigen measurements across trials for the respective patient group (seropositive, seronegative); these correspond to the locations of the internal knots for the restricted cubic splines. The dotted lines represent the 10th and 90th percentiles of antigen measurements in the respective group.

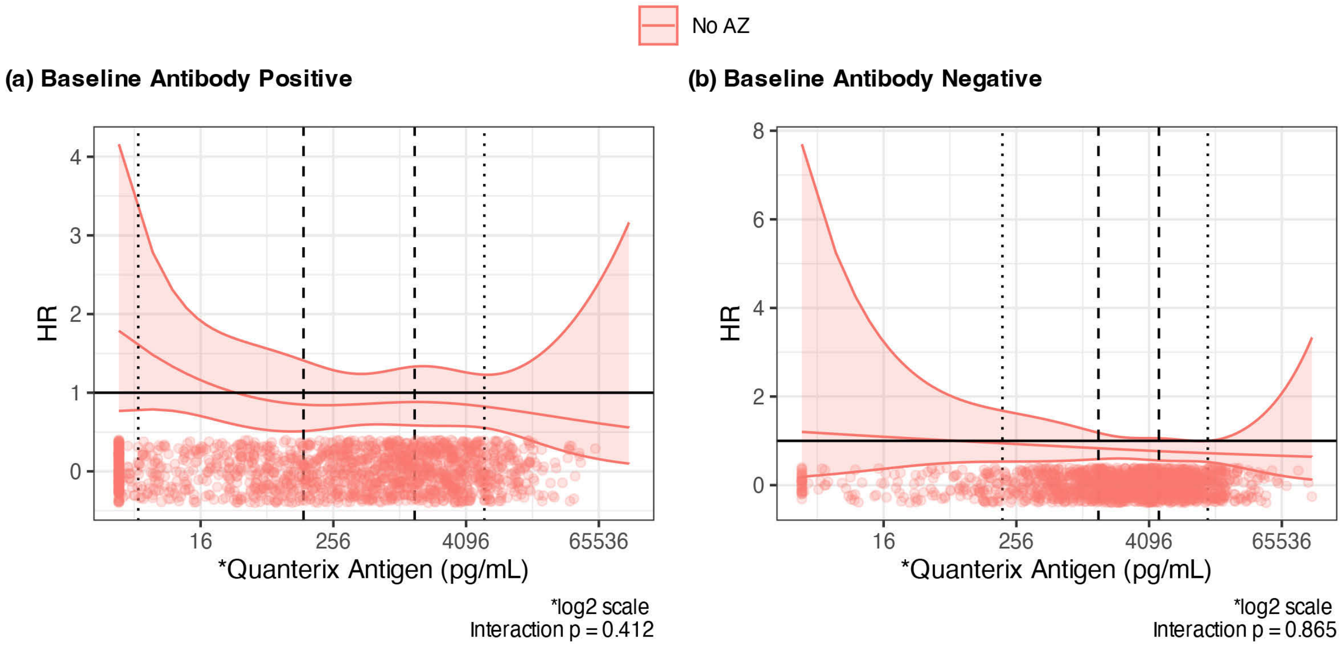

Supplement: S18 Fig — The points show the observed distribution of baseline antigen measurements in patients. The dashed lines represent the 33rd and 66th percentiles of antigen measurements across trials for the respective patient group (seropositive, seronegative); these correspond to the locations of the internal knots for the restricted cubic splines. The dotted lines represent the 10th and 90th percentiles of antigen measurements in the respective group. (PDF) [file pmed.1004616.s018.pdf]
